# Patients’ perspectives on bronchiectasis: findings from a social media listening study

**DOI:** 10.1183/23120541.00096-2021

**Published:** 2021-08-02

**Authors:** Irisz Delestre-Levai, Stefano Aliberti, Marta Almagro, Chiara Carnini, James D. Chalmers, Sharath C. George, Soumya Shukla, Alan Timothy, Maria Carmela De Vuono

**Affiliations:** 1 Chiesi Farmaceutici SpA, Parma, Italy; 2 Respiratory Unit and Cystic Fibrosis Adult Center, Internal Medicine Dept, Fondazione IRCCS Cà Granda Ospedale Maggiore Policlinico, Milan, Italy; 3 Dept of Pathophysiology and Transplantation, University of Milan, Milan, Italy; 4 EMBARC/ELF Bronchiectasis Patient Advisory Group, Sheffield, UK; 5 School of Medicine, University of Dundee, Ninewells Hospital and Medical School, Dundee, UK; 6 Decision Resources Group, Part of Clarivate, London, UK

## Abstract

Although it is of great importance for healthcare professionals to ensure that patients’ needs and concerns are valued and that they feel confident in the quality of the care they receive, there have been few studies specifically addressing the opinions, experiences and needs of patients with bronchiectasis, and more importantly the emotional impact of the disease, diagnosis and treatment.

Using enterprise grade social listening tools, a comprehensive search around bronchiectasis was performed in five languages, on different social media platforms between January 2018 and December 2019 to obtain the perspectives of patients and caregivers from nine countries on symptoms, treatments and burden of the disease.

Over 27 000 mentions of bronchiectasis were identified on social media channels, 38.8% of which were posted by patients and caregivers. Approximately 1600 posts were found on bronchiectasis symptoms, out of which persistent cough, shortness of breath and mucus production (22%, 20% and 18%, respectively) were the most commonly discussed. The research revealed that existing diagnostic tests often delay diagnosis or provide inaccurate results, leading to multiple rounds of consults and substantial delays in treatment initiation and management of the disease. Misdiagnosis was common across different age groups, especially among patients without severe symptoms, and this was associated with an emotional burden of anger, confusion, frustration and anxiety.

Analysis of social media presents a new approach to derive insights on patients’ experiences and emotions with bronchiectasis and has the potential to complement more traditional approaches to drive more patient-focused drug development.

## Abstract 2

**Patients’ journey in bronchiectasis from symptoms and diagnosis to everyday life with the disease described for the first time through social media listening, a novel approach to gather unbiased insights into patients’ experiences and emotions** https://bit.ly/3yFLG5v

## Introduction

Bronchiectasis is a chronic respiratory disease defined by abnormal and irreversible dilatation of the bronchi. The disease can be caused by many different aetiologies, and it is clinically characterised by a variety of symptoms, including cough, sputum production and airway infection, and can often present with recurrent exacerbations [1]. bronchiectasis has been a neglected area of research in respiratory medicine for decades despite its high morbidity and associated mortality across all ages [2]. Nowadays bronchiectasis is seen as a distinct, although heterogeneous condition in its own right [3] and is receiving much more attention.

Although it is of great importance for all healthcare professionals to make sure that the patient's values, needs and concerns are valued at all stages of treatment and that patients feel confident in the quality of the care they receive, there have been only a very few studies specifically addressing the opinions, experiences and needs of patients with bronchiectasis.

A novel approach to listen to the voice of patients is represented by social media listening (SML): conversations on social media can offer unprecedented insights on how patients live with their disease and how they think about their condition. Nowadays patients do not only use internet as a source of information but also to exchange information with other users leveraging on platforms like Facebook, forums or blogs. The online data volume has been getting bigger exponentially in recent years, making social media one of the largest, most diverse and most valuable sources of information available [4]. SML offers access to genuine opinions, emotions and comments: as the internet offers anonymity, patients are more willing to share their experiences, fears, concerns and challenges with others with the same condition [4].

This is the first study using SML to understand patients’ experience of living with bronchiectasis. We hereby give an insight into the patients’ emotional journey from symptoms and diagnosis to everyday life with the disease from a unique and unbiased perspective: that of the patients.

## Material and methods

### Study design and data source

A comprehensive search around bronchiectasis was performed on different open social media platforms (*e.g.* Twitter, Instagram, YouTube, as well as blogs, news, forums and public Facebook pages or groups) in English, Spanish, French, Italian and German languages to obtain patients’ and caregivers’ perspective on symptoms, treatments and burdens of the disease. Social media posts between January 2018 and December 2019 from the United Kingdom (UK), Spain, France, Italy, Germany, the United States (US), Canada, Australia and New Zealand were retrieved using enterprise grade social listening tools. Bronchiectasis-related posts and conversations were extracted using the following predefined search terms: “Bronchiectasis” or “Non-cystic fibrosis bronchiectasis” or “Non-CF Bronchiectasis” or “NCFBE”. These key words were combined with other search terms related to symptoms, disease severity and diagnosis (supplementary table S1).

Social media contents selected for the study include posts by probable patients (who either had received a confirmed diagnosis, were waiting for a diagnosis or had been misdiagnosed) and by probable caregivers.

### Selection of posts

Keywords and phrases related to bronchiectasis or non-cystic fibrosis bronchiectasis in reference to lung conditions along with their misspellings, abbreviations and synonyms were used to query a 2-year historical database of social media conversations.

The keywords set was refined in an iterative process, starting from broader keywords and refining them further to reduce noise and irrelevant mentions. Moreover, the dataset was cleaned to ensure that it included only posts and responses of probable patients or probable caregivers (detailed in the supplementary material).

### Ethical considerations

All data analysed in this study were obtained from publicly accessible sources without accessing password-protected information and in compliance with applicable data protection laws. All data presented within this study have been aggregated and anonymised and do not include any personally identifiable information (PII).

### Categorisation and indexing of posts

First-person mentions of the disease or treatment were automatically classified as patient conversations, whereas third person references to the disease experiences of a child, parent or family member, or their care were identified as caregiver conversations. This classification was further validated and checked by human analysts to ensure accuracy.

Passive conversations like retweets and shares of popular content were not included in the analysis of first and second person mentions.

Patient journey stages (symptoms, diagnosis, treatment) were identified using predefined taxonomies of keywords and expressions, and further analysed for concerns expressed by patients and caregivers. Conversations in each stage were examined by analysts and classified into pain points and needs expressed by patients, as well as decision points and drivers of discussion (detailed in the supplementary material).

## Results

Of the total 61 694 posts retrieved, 34 000 were excluded as they were considered irrelevant mentions. The most common source of the remaining 27 694 bronchiectasis posts was Twitter (51%), followed by other channels, *e.g.*Instagram, YouTube, as well as blogs and forums.

**FIGURE 1:**
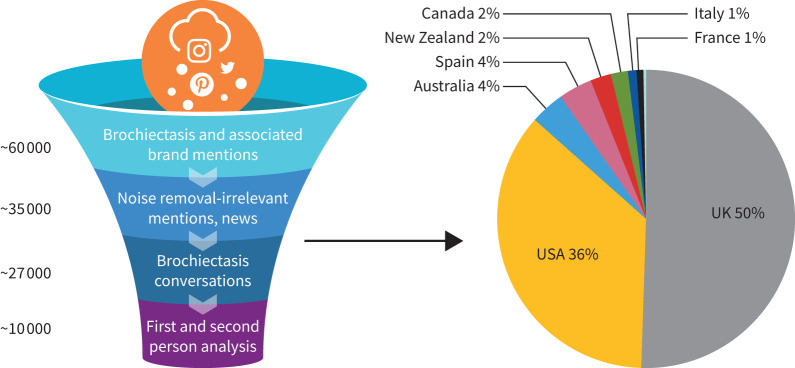
Volume of bronchiectasis mentions across geographies. n=27 694.

The UK and the US were regions with the majority of mentions (86% cumulatively) around the disease followed by Australia and Spain, which contributed to 4% of overall disease mentions in each region (figure 1).

Overall, 10 770 out of 27 694 were identified as posts from patients or caregivers and selected for the study, the remaining were news on research updates, fundraisers and general disease awareness-related posts from other stakeholders including advocacy groups and industry experts. Of the selected conversations, 90% were from patients and 10% were from caregivers.

Twitter emerged as the key channel with 78% of conversations among the bronchiectasis patient and caregiver community. Supplementary table S2 provides an overview of popular channels of conversation among patients and caregivers discussing bronchiectasis, while supplementary table S3 elaborates the share of patient and caregiver conversations from various regions.

### Symptoms

Based on 1600 total mentions of bronchiectasis symptoms, persistent cough, shortness of breath and mucus production (22%, 20% and 18% of mentions, respectively) were found to be the most commonly discussed symptoms among patients online. Shortness of breath was found to be one of the critical symptoms to aid diagnosis, while persistent cough and mucus in lungs led to inconclusive diagnosis.

The discussions around symptoms not only concern patients who have been living with the disease for quite some time, but also those who are new to the condition or have previously been misdiagnosed. Although mentions of clubbing, presence of blood in sputum and weight loss were low (5%, 3% and 2% of mentions, respectively), these symptoms were reportedly crucial in raising suspicion of bronchiectasis.

### Diagnosis

Across all studied geographies, 128 patient and caregiver conversations raised concerns about the accuracy of existing diagnostic tests (*e.g.* radiographs, pulmonary function tests, bronchoscopy), which could potentially result in inaccurate or inconclusive results leading to multiple rounds of consults and consequently causing delays in the treatment initiation and management of the disease. In addition, 328 patient conversations reported experience regarding misdiagnosis that led to worsening of their symptoms.

Some aspects, however, seemed to be country specific: in France, patients discussed the absence of specialist diagnostic centres; in Canada, the insurance coverage for the diagnostic tests is considered a major problem; in Australia, patients seem not to have access to noninvasive tests for detecting bronchiectasis; and in the UK, patients complained about the difficulty of getting a specialist appointment in the National Health Service (NHS). Moreover, in Germany, patients perceived a lack of knowledge among healthcare professionals (HCPs) regarding the detection of bronchiectasis, and in the US, the pulmonologist's and the radiologist's opinion seem to differ when it comes to determining the diagnosis.

The results of the study indicate that patients are likely to get a confirmed diagnosis at a later stage of the disease due to inappropriate or delayed diagnosis.

Based on a sample of conversations (n=50) where age was revealed by the commenter, it was noted that a high number of patients (∼46%) discussed being diagnosed when they were young adults, while conversations around diagnosis of children with bronchiectasis was less discussed online.

Moreover, based on 957 mentions, 46% were posts from patients who presented to their HCP with symptoms such as chronic cough, haemoptysis, shortness of breath and wheezing and were misdiagnosed with asthma and/or COPD. Moreover, 20% of patient conversations suggested that they remained undiagnosed and were requested to undergo further assessments, and 34% of these discussions indicated that patients were diagnosed as having bronchiectasis as a consequence of other condition(s).

### Treatment

#### Bronchiectasis treatment journey

Observations from social media data suggested that treatments are likely to be chosen by HCPs based on guideline recommendations. In addition, the physician's experience in treating patients with bronchiectasis plays an important role as well. Patients who are suspected of having bronchiectasis are generally prescribed treatments to manage symptoms (*e.g.* cough, blood in sputum), and those living with comorbidities such as asthma or COPD are advised to solely continue with the treatments they receive for their obstructive respiratory condition. On the other hand, patients whose diagnosis is not confirmed frequently seek advice on online forums and consult with multiple specialists in the hope of finding a cure for the physical and psychosocial obstacles they are facing on a daily basis.

**FIGURE 2:**
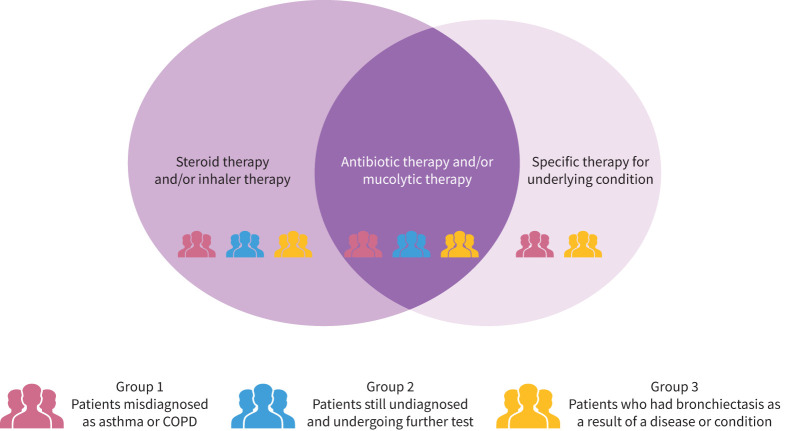
Treatment regimen for bronchiectasis patients.

The Venn diagram in figure 2 represents the treatment regimens that patients are often prescribed during exacerbation. The size of the circle correlates with the number of mentions of the different treatments. According to the mentions, all patients are given steroid/inhaler therapy to control breathing symptoms like cough and wheezing. Antibiotic and/or mucolytic therapies are prescribed in order to combat associated infections and to reduce mucus production, respectively. There were no mentions of any treatments that are specific to bronchiectasis.

Patients are categorised in each of these groups based on their conversations and experiences shared online.

#### Treatment-related side-effects

Very few patients (∼2.5%) shared their experience online related to side-effects associated with treatment.

The side-effects reported were: risk of infection (n=64) and diabetes (n=16) related to steroid therapy; acid reflux (n=6) and tremor (n=10) related to inhaler therapy; allergic reaction (n=26) and neuropathy (n=3) associated with antibiotics; and dryness of mouth (n=3) and sore throat (n=16) linked to other therapies (*e.g.* mucolytics).

#### Antibiotic treatment and airway clearance devices

Conversations around antibiotic treatment were limited constituting ∼12% of all patient and caregiver conversations (detailed in the supplementary material). Although antibiotic therapy helps in managing severe bronchiectasis symptoms, patients show apprehension towards the long-term use of them. The research revealed that patients feel exhausted from being on antibiotics for prolonged periods (n=235), and they fear that long-term use poses a threat of resistance and low tolerability that could potentially lead to further difficulties in managing their condition (n=54). They also find it extremely burdensome having to provide sputum samples on a regular basis in order to guide the decision making in relation to selecting the most suitable antibiotic option (n=65).

In total, 150 conversations were identified as discussions during the early treatment phase and use of antibiotic therapy during re-infection or post-surgery phase. Twenty-three per cent of these conversations discussed side-effects concerning prolonged use of treatment, while 18% of the discussions captured among patients with recurrent infections and/or in post-surgical phase were speculating that the potential reason for the deterioration of their condition may be due to having refused antibiotic treatment earlier on. Other topics of discussions with a lower number of mentions included switching medication for better tolerability (19% of conversations) and allergy to antibiotics reducing treatment options (14% of these conversations).

The study also revealed that patients often seek advice from each other on their experience of managing excessive mucus with airway clearance vests and apparatus. Those who are new to these often look for tutorials and practical advice on how to clean and maintain them. A few discussions were captured on airway clearance device availability in the market and their key features. Although low (<50 mentions), some patient conversations expressed concern about the prescription, availability and cost of these devices as well as oral thrush due to the use of inhalers. Inhalers and airway clearance devices are considered unaffordable for those with limited or no insurance cover. Moreover, delays due to insurance approval also have a significant negative impact on patients. It was reported that a battery-operated portable device may contribute to better compliance as it has the great advantage of giving patients the freedom to move around during the session. The management of infections such as oral thrush caused by the usage of spacers along with inhalers was also discussed.

### Living with bronchiectasis

Overall, there were 3500 mentions related to the experience of living with bronchiectasis. Based on the nature of the conversations, two main topics were identified, one related to emotions and one related to awareness and knowledge.

#### Emotions

Conversations were analysed for emotional impact of the condition on commenters. Over 2500 disease mentions from patients were identified for this analysis.

**FIGURE 3:**
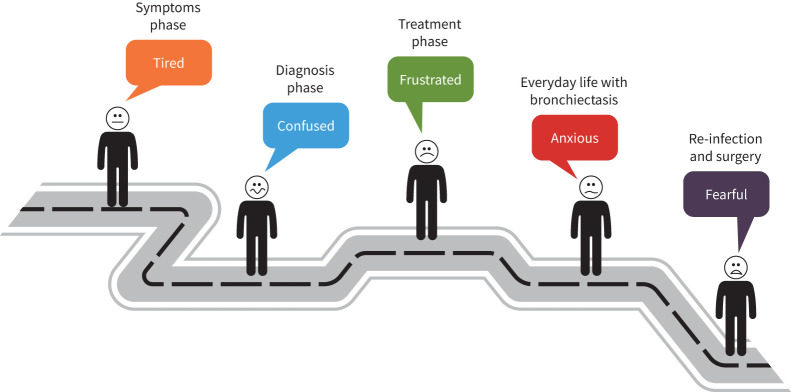
The emotional journey of bronchiectasis.

The experience of living with bronchiectasis has been recognised as a source of various emotions (figure 3).

The results of our research show that the process leading to the diagnosis of bronchiectasis is often very complicated, mainly due to the commonality of symptoms with other diseases; patients struggle with daily symptoms and respiratory infections for years before being diagnosed. Patients are tired of waiting months or even years for a correct diagnosis during which time they are subject to several specialists’ consultations often without a conclusive result (n=213). They feel confused about the process related to diagnosis (n=420) and frustrated about not receiving the right treatment (n=342). Once diagnosed, fear of recurrent respiratory infections, side-effects of treatments and uncertainty regarding future life with the condition were the most prominent emotions discussed by patients (n=309). Embarrassment and frustration were commonly associated with the difficulties in accomplishing simple activities and daily tasks (n=544).

#### Awareness and knowledge

Patients’ experience of living with the condition is also shaped by the need to seek and generate awareness and knowledge around their condition and to share their own experiences with other people. Patients turn to peers for support through online forums and offline support groups. They primarily seek advice from peers as they learn about the condition followed by support from caregivers and physicians.

In total, 942 conversations revealed that people are focused on trying to maintain a normal life while managing bronchiectasis and hence continuously seek information on how to manage and improve their quality of life. They strongly believe in modifying their lifestyle along with exercise and healthy diet, because this may play an important role in their daily quality of life.

## Discussion

The widespread involvement of patients, families and caregivers is fundamental to the delivery of appropriate, meaningful and safe healthcare and is essential at every stage of the care cycle including drug development and regulatory decision making [5]. Collecting data from patients provides important insights into the most significant symptoms of their disease and their impact on daily life. Traditionally, approaches such as conducting face-to-face interviews, surveys or focus group discussions have been used to gather information on the patient's experiences and perspectives on their condition [6]; however, technology advancements have revolutionised the communication channels which now offer opportunities for improving medical care, often at a faster pace. Patients are more and more active online and use social media to share experiences, debate healthcare practice issues, discuss treatment options and search for healthcare professionals as well as to participate and express themselves openly [7, 8]. In addition, the advocacy groups are increasingly empowered to contribute to this novel approach, and social media offer the means for directly engaging patients to obtain input and perspectives.

An open-source social media search may provide a valuable tool in assessing patient perspectives on a disease and therefore offers an innovative approach to collecting patient experience data. Patients have a unique ability to contribute to the understanding of the broader context of their disease, which is becoming increasingly important to all who care for them and to all facing the many challenges of the drug development process. The patient-led model of research represents a step forward in healthcare as it recognises the value in patient involvement at every stage of clinical research [9, 10].

The strengths of SML methodology can be identified in the authentic and unbiased insight emerging from patients’ conversations. The thoughts and opinions expressed openly by patients online distinguish this approach from traditional, structured and solicited patient research, and it is considered that the results of such SML studies should reflect spontaneous patient perspectives on their disease, their unmet needs and the emotional burden using their natural language [11, 12]. In the case of SML on bronchiectasis conversations, it is important to highlight that the outcomes of the analysis are in line with patient registry data. The EMBARC (European Multicentre Bronchiectasis Audit and Research Collaboration) registry confirms that congestion, cough, sputum production and shortness of breath are the most burdensome symptoms affecting up to 90% of bronchiectasis patients [13]. These symptoms, together with exacerbations and tiredness, represent aspects of bronchiectasis found either difficult or very difficult to manage by patients [3].

Owing to the interactive nature and given the popularity of social media across different geographies, SML can also provide the ability to research and analyse the perspectives of large groups of patients across different countries and languages, which would be unfeasible with traditional research [14, 15].

The main limitations of SML are related to the quality and reliability of the conversations, which may affect accuracy. We cannot confirm with certainty whether all patients have clinically verified diagnoses or are self-diagnosed [15]. Another limitation is that not all patients are equally likely to write about their medical experiences online. As a result, we may only have the opportunity to view and analyse the experiences of a certain segment of the population [15]. Some social media channels investigated in this study are particularly popular and often only used in certain countries (*e.g.* the US). This could represent a limitation of the present study, and therefore caution must be applied, as the findings might not be translated to other countries [16, 17].

Additionally, negative conversations may be vocalised more often than positive perceptions/experiences [11]. However, in our study, we examined >27 000 mentions of bronchiectasis identified on several social media channels, which should allow for discussions and conversations from a variety of different communities, individuals and age groups allowing the mitigation of these limitations.

In summary, patients with bronchiectasis actively share experiences on forums and seek information online through social media. SML presents a new method to derive insights on patients’ experiences with bronchiectasis and has the potential to complement traditional approaches to drive more patient-focused drug development. The results of our research indicate that from the patients’ perspective, relief from cough, shortness of breath and mucus production would possibly be the most valuable aspects of disease management in bronchiectasis. In addition, increased awareness among physicians about the tests and procedures in bronchiectasis would be fundamental to timely diagnose the condition and provide appropriate care and support to the patients.

A key element of novelty provided by this SML study is the possibility of mapping the emotional journey of bronchiectasis patients from the start of symptoms onward: this type of analysis provides an understanding of how the predominant emotions are evolving along with the disease stages, and at what stage the emotional challenges are the highest. Emotional journey analysis gives us an opportunity to gain a richer picture of the patient, understand the potential factors that could lead to an improved quality of life and the potential drivers and barriers to proper disease management adoption.

The findings from qualitative SML studies should be interpreted with caution considering that the quality and reliability of the conversations cannot be verified and therefore the accuracy of the information shared is not guaranteed. Nevertheless, the importance of such studies and in particular the recognition of patients’ perspective as a source of information that allows HCPs to serve their patients’ multiple and complex needs, cannot be emphasised enough.

## Supplementary material

10.1183/23120541.00096-2021.Supp1 **Please note:** supplementary material is not edited by the Editorial Office, and is uploaded as it has been supplied by the author.
Supplementary material 00096-2021.supplement

## References

[1] Polverino E, Goeminne PC, McDonnell MJ, et al. European Respiratory Society guidelines for the management of adult bronchiectasis. Eur Respir J 2017; 50: 1700629. doi:10.1183/13993003.00629-2017 28889110

[2] Regan KH, Hill AT. Emerging therapies in adult and paediatric bronchiectasis. Respirology 2018; 23: 1127–1137. doi:10.1111/resp.13407 30242794

[3] Aliberti S, Lonni S, Dore S, et al. Clinical phenotypes in adult patients with bronchiectasis. Eur Respir J 2016; 47: 1113–1122. doi:10.1183/13993003.01899-2015 26846833

[4] Mikolajczak C. Social media listening: uncovering patients’ needs. www.iqvia.com/-/media/iqvia/pdfs/cese/germany/news/iqvia-social-media-listening-artikel-2017.pdf?la=de-de&hash=C5EE8FCE73016BB6C1C6E78DE83C7561 Date last updated: 28 July 2017. Date last accessed: 4 November 2020.

[5] Food and Drug Administration. Patient-focused drug development: collecting comprehensive and representative input: draft guidance for industry, food and drug administration staff, and other stakeholders. 2018. www.fda.gov/ucm/groups/fdagov-public/@fdagov-drugs-gen/documents/document/ucm610442.pdf Date last updated: 16 June 2018. Date last accessed: 4 November 2020.

[6] LaVela SL, Gallan A. Evaluation and measurement of patient experience. Patient Exp J 2014; 1: 28–36. doi:10.1177/237437431400100206

[7] De Martino I, D'Apolito R, McLawhorn AS, et al. Social media for patients: benefits and drawbacks. Curr Rev Musculoskelet Med 2017; 10: 141–145. doi:10.1007/s12178-017-9394-7 28110391 PMC5344865

[8] Rozenblum R, Greaves F, Bates DW. The role of social media around patient experience and engagement. BMJ Qual Saf 2017; 26: 845–848. doi:10.1136/bmjqs-2017-006457 28428244

[9] Sacristán JA, Aguarón A, Avendaño-Solá C, et al. Patient involvement in clinical research: why, when, and how. Patient Prefer Adherence 2016; 10: 631. doi:10.2147/PPA.S104259 27175063 PMC4854260

[10] Longtin Y, Sax H, Leape LL, et al. Patient participation: current knowledge and applicability to patient safety. Mayo Clin Proc 2010; 85: 53–62. 20042562 10.4065/mcp.2009.0248 PMC2800278

[11] Cook N, Mullins A, Gautam R, et al. Evaluating patient experiences in dry eye disease through social media listening research. Ophthalmol Ther 2019; 8: 407–420. doi:10.1007/s40123-019-0188-4 31161531 PMC6692792

[12] McGoon MD, Ferrari P, Armstrong I, et al. The importance of patient perspectives in pulmonary hypertension. Eur Respir J 2019; 53: 1801919. doi:10.1183/13993003.01919-2018 30545977 PMC6351339

[13] Polverino E, Blasi F, Ringshausen F, et al. Determinants of quality of life in bronchiectasis using the quality of life bronchiectasis (QOL-B) questionnaire: data from the EMBARC registry. Eur Respir J 2018; 52: OA4951.

[14] Cook NS, Kostikas K, Gruenberger J-B, et al. Patients’ perspectives on COPD: findings from a social media listening study. ERJ Open Res 2019; 5: 00128–02018. 10.1183/23120541.00128-2018 PMC6368996 30775374

[15] Keller MS, Mosadeghi S, Cohen ER, et al. Reproductive health and medication concerns for patients with inflammatory bowel disease: thematic and quantitative analysis using social listening. J Med Internet Res 2018; 20: e206. doi:10.2196/jmir.9870 29891471 PMC6018236

[16] Ortiz-Ospina E. “The rise of social media”. 2019. Available from: https://ourworldindata.org/rise-of-social-media#licence Date last updated: 18 September 2019. Date last accessed: 22 December 2020.

[17] Perrin A, Anderson M, Pew Research Center. Share of U.S. adults using social media, including Facebook, is mostly unchanged since 2018. 10 April 2019. https://pewrsr.ch/2VxJuJ3 Date last updated: 10 April 2019. Date last accessed: 10 November 2020

