# Supplementary material for: Patients’ perspectives on bronchiectasis: findings from a social media listening study
Source: ERJ Open Res. 2021 Aug 2;7(3):00096-2021. doi: 10.1183/23120541.00096-2021 (PMC8326711; doi:10.1183/23120541.00096-2021)
Supplement: Supplementary file 1 [file 00096-2021.supplement.pdf]

CONTENT

METHODS: Data Cleaning

TABLE S1: Search Terms

METHODS: Categorisation and indexing of posts

TABLE S2: RESULTS: Key social channels in the study design

TABLE S3: RESULTS: Patient and Caregiver conversations across regions

RESULTS: Antibiotics

Methods: Data Cleaning

Social media posts were collected after a comprehensive search across social media platforms. This data was downloaded in a Microsoft excel and was cleaned to remove spam and duplicates (based on social media link and/or content).

The process of spam removal was essential to eliminate posts which were not relevant to the scope of this study (i.e. social media posts around buying and selling of drugs by online retailers and content leading to market research or market forecasts available for pharma companies). Industry news and updates were also excluded.

Several misspellings and hashtags were also used in the data aggregation process to account for social media texts which are written casually and include disease hashtags for better online reach. It might be possible that some online content was missed during the search due to incorrect spelling of the online posts.

Posts clearly mentioning CF-associated bronchiectasis were excluded from the study. Nevertheless, due to the qualitative nature of this type of analysis uncertainty about the reliability of the declared diagnosis still represents a potential limitation.

Table S1:

Search terms:

The keywords noted in the table below were translated into local languages to provide better coverage in European regions.

**Disease Related**	**Symptom Related**	**Diagnosis Related/Other**
Bronchiectasis	Cough	Chest-X Ray
Non-cystic fibrosis bronchiectasis	Wheezing	Computerized tomography (CT) scan
Non-CF Bronchiectasis	Fatigue	Blood tests
Non cystic fibrosis bronchiectasis	Clubbing	Lung function tests (LFT)
NCFBE	Respiratory infection	Pulmonary function tests (PFT)
	Breathlessness	Bronchoscopy
	Shortness of breath	Pulmonologist
	Mucus	Antibiotic
	Sputum	Mucolytic
	Chest pain	Airway clearance
	Coughing up blood	Device
	Weight loss	Inhaler

NCFBE: Non-Cystic Fibrosis Bronchiectasis

Methods: Categorisation and indexing of posts

Each post was reviewed to evaluate the mention of different parameters. Among those selected for the study, not every post included all parameters such as symptoms, treatment, severity, diagnosis, etc. However, each post has a mention of one or more parameters.

Demographic information about the users (sex, age and time since diagnosis) was collected only when mentioned in the text. No background information was collected from the studied data.

Table S2:

Results: Key social channels in the study design

**Channel**	**Share of voice among patient and caregiver conversations**<br><br>*Total included posts: N=10,770*
Twitter	78%
Forums and Reddit	8%
Blogs/ YouTube	4%
Others	10%

Table S3:

Results: Patient and Caregiver conversations across regions

**Country**	**Volume of patient and caregiver mentions**<br>*Total included posts: N=10,770*
United Kingdom	5,384
United States of America	4,807
Australia	258
Italy	130
France	55
Canada	48
Spain	42
Germany	31
New Zealand	15

Results: Antibiotics

Among various antibiotics discussed online, conversations around Azithromycin (N= 348) were the most prominent as patients and caregivers discussed efficacy and their experiences as well as ongoing research about the drug. Other key antibiotic therapies discussed online included doxycycline, amoxicillin and

ciprofloxacin. The majority of the conversations (N= 734) did not specify the type of antibiotic the commenters had been prescribed or treated with and the comments remained generic.
